# Pharmacist Interventions in Minimizing Drug Related Problems in Diabetes With Co-Existing Hypertension: A Five-Year Overview and Ground Report From India

**DOI:** 10.3389/ijph.2023.1605808

**Published:** 2023-04-03

**Authors:** Ian Osoro, Mohammed Amir, Manisha Vohra, Amit Sharma

**Affiliations:** Department of Pharmacy Practice, ISF College of Pharmacy, Moga, India

**Keywords:** diabetes, hypertension, adverse drug reactions, length of stay, drug interactions

## Abstract

**Objective:** The study aimed to investigate the pharmacist interventions in minimizing drug-related problems in diabetes with co-existing hypertension.

**Methods:** Prospective observational study.

**Results:** Overall, a total of 628 interventions were recommended for 1,914 patients during the 5-year period of study. Among all the interventions, the majority were suggested regarding “substituting the drug” (39%), change in frequency of administration (25%), and addition of drug (14%). Patient compliance status was found significant (*p =* 0.29 ± 0.07).

**Conclusion:** Clinical pharmacists have a crucial role in minimizing drug related problems. Particularly, there should be a greater emphasis on patient counselling and patient follow-up.

## Introduction

Patient safety is the priority of all healthcare workers. However, the treatment and management of co-morbid conditions has proven to be challenging even to the healthcare professionals. This is because the available clinical guidelines are mainly based on the assumption that patients suffer from a single disease (1). Diabetes co-existing with hypertension (HTN) is a very common occurrence especially in type 2 DM (Diabetes Mellitus) patients. According to The Global Burden of Disease study HTN and DM have been identified to be the leading cause of untimely deaths and disability globally (2).

It is globally estimated that the prevalence of type 2 diabetes mellitus (T2DM) will be 366 million by 2030 whereas that of hypertension will be 1.56 billion adults by 2025 (3, 4). The prevalence of hypertension in diabetic patients is almost double that of non-diabetic and the patients with hypertension as a comorbidity are at a higher risk of mortality and cardiovascular disease development (5, 6). A study by Geldsetzer et al (7) which involved 1.3 million adults in India revealed that this comorbid condition was more prevalent in the middle and old age. Having a team approach (i.e., physicians, nurses, clinical pharmacists, dieticians, etc.) in the therapy of DM with HTN patients has shown to be effective (8). Managing the blood pressure levels in diabetic patients, adherence to both pharmacological and non-pharmacological treatments such as exercising, etc., and use of proper medication with minimum adverse effects are some of the methods that can be used to reduce the risk of developing hypertension in diabetic patients.

Clinical pharmacists are specially trained personnel on pharmacotherapy and their major role in healthcare setting is to ensure safe, effective, and judicious use of medications in the patients (9). They identify drug related problems (DRPs) which are recurrent in hospitals particularly during prescribing, dispensing, administration and adherence of the drugs (10). A drug related problem is a negative outcome of a medication administered to a patient and fortunately, they are mainly preventable. Clinical pharmacists have been encouraged to use the SOAP (Subjective, objective, assessment, plan) or FARM (Findings, assessment of findings, resolution of the problems and monitoring) progress note in order to easily identify and report DRPs (11, 12). Common outcomes of DRPs in DM with HTN patients include: decreased quality of life, longer hospital stays, and increased costs (13).

The inclusion of clinical pharmacists in the healthcare team helps address the challenges related to DRPs as they offer valuable interventions on case-by-case basis. A randomized clinical trial study showed that the addition of clinical pharmacist to the healthcare team resulted in decreased mortality and healthcare costs. The pharmacist interventions were on the drug selection, dosages and monitoring needs during the study (14). Additionally, patient counselling and follow-up is another common pharmacist intervention. Clinical pharmacists have therefore become an integral part of quality healthcare delivery systems even as more emphasis is being laid on team care-based model (15, 16).

The absence of long-term published describing the clinical pharmacists’ interventions in India was the rationale for conducting this study. Therefore, the main objective of this study was to investigate the pharmacist interventions in minimizing drug-related problems in diabetes with co-existing hypertension in the medicine departments of three hospitals in Punjab, India.

## Methods

### Setting

In this observational study, all the patients referred to the medicine department of the three different hospitals of Moga, city located in Punjab state of India (17, 18) were enrolled in the study.

### Study Design and Patient Selection

For this multi-centre prospective study, patients were included from November 2015 to December 2020. In the present study, all the interventions of clinical pharmacists made over 5 years were included. The present study is part of a completed research entitled “drug utilization study of diabetes and hypertension at tertiary care hospital.” The population size of the Moga city was found N = 2.98 lakhs (17). The sample size is calculated with the ‘Epi Info’ software (19, 20). A total of 2,622 patients suffering from diabetes mellitus (D.M.) and hypertension (HTN) were screened during the study period. Out of the 2,622 patients, 1,914 patients were enrolled in the statistical analysis. A total of 708 patients were excluded while analysing data because of missing value; some patients were lost during the follow-up. Patients diagnosed with diabetes mellitus and hypertension with or without complications admitted to (IPD) in-patient department of the hospital were assessed. The study’s inclusion criteria include patients visiting the hospital for follow-up, both genders with age >18 years, diabetes with co-existing hypertension patients and those that were willing to participate. Institutional Ethics Committee (IEC-ISFCP, Moga) ISF College of Pharmacy, Moga, Punjab approved the study (Ref. No. ECR/296/Indt/PB/2017/ISFCP/136). The confidence interval of the study was chosen as 97% (21). All statistical tests were carried out at the two-sided 3% significance level by statistical analysis software SPSS ver. 25.

### Data collection

**FIGURE 1:**
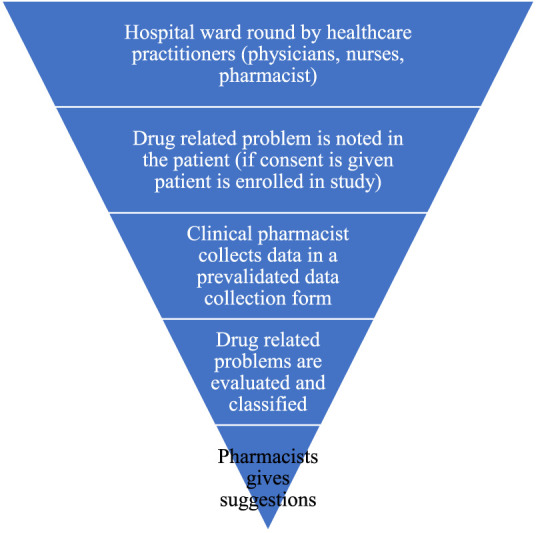
Description of the data collection procedure (Drug utilization study of diabetes and hypertension at tertiary care hospitals, India, five years study, 2020).

Figure 1 gives a simple flow of how data was collected in our study. A pre-validated data collection form which includes a questionnaire containing 105 variables was used to collect data after obtaining consent from the patient. The variables include: Age, gender, type of ADRs, type of drug interactions, type of drug related problem, type of pharmacist intervention, cost, length of hospital stay. All study costs were recorded in INR, and pharmacoeconomic variables were converted to USD (INR: 73.36 = 1 USD).

The mean age of the patients suffering from diabetes mellitus (type-I and type-II) with co-existing hypertension (µ) and standard deviation (S.D.) was found to be (M = 53.85, SD = 11.54) years. The normality test was performed, which was found normally distributed Kolmogorov-Smirnov and Shapiro-Wilk (*p* = 0.36 and 0.223), respectively (22–24).

### Ethics Approval

The present study was approved by IEC of ISF College of Pharmacy, Moga, Punjab (Ref. No. ECR/296/Indt/PB/2017/ISFCP/136).

## Results

Out of 1,914 patients, 914 were male (47.8%) and the females were 1,000 (53.65%).

During the study, 63 different types of ADRs were detected by the clinical pharmacist in 628 (32.8%) of the patients. The majority of the patients were suffering from adverse drug reactions like hypotension (3.3%), hypoglycaemia (3.2%), mild itching or rash (1.5%), swelling in their feet or ankles, severe drowsiness (1.4%) followed by skin rashes (1.0%). In 1,914 patients, 42 different kinds of drug interactions were observed in 1,612 cases. The majority of the drug interactions were seen between Insulin + Emeril 226 (11.8%) followed by 111 (5.8%) Emeril + PCM + Diclofenac. The drug-related problems include inappropriate drug form (6.1%), inappropriate drug (4.6%), no clear indication for drug use (4.6%), contraindication for the drug (4.1%), no drug prescribed but clear indication (3.8%), and duplication of a therapeutic group or active ingredient (1.5%).

Additionally, change in frequency of administration was found to be 153 (8.0%), addition of drug 89 (4.6%), change in the duration of therapy 50 (2.6%) and change in drug dose 33 (1.7%). Pharmaceutical aid was included in 22 (1.1%) of the cases.

Tables 1, 2 below demonstrates the characteristics of the drug prescriptions and types of drug related problems noted and their statistics (Tables 3, 4).

**TABLE 1:** Characteristics of the drug prescriptions (Drug utilization study of diabetes and hypertension at tertiary care hospitals, India, five years study, 2020).

Number of drugs during discharge	Frequency	Percent
2–4 drugs including a combination	73	3.8
5–8 drugs including a combination	853	44.6
>8 drugs including a combination	988	51.6
Total	1,914	100.0
**Number of antibiotics prescribed**	**Frequency**	**Percent**
No antibiotics prescribed	307	16.0
Single antibiotic	1,289	67.3
Two antibiotics combination	194	10.1
Three antibiotics combination	71	3.7
Four antibiotics combination	53	2.8
Total	1,914	100.0
**Number of injections and IV infusions prescribed**	**Frequency**	**Percent**
No injections prescribed	21	1.1
One injection only	336	17.6
Two injection	962	50.3
Three injection	504	26.3
Four injection	80	4.2
Five injection	11	0.6
Total	1,914	100.0
**Number of drugs prescribed from EDL**	**Frequency**	**Percent**
No drugs from EDL	23	1.2
One drug prescribed from EDL	364	19.0
Two drugs prescribed from EDL	795	41.5
Three drugs prescribed from EDL	475	24.8
Four drugs prescribed from EDL	202	10.6
Five drugs prescribed from EDL	55	2.9
Total	1,914	100.0

Table 1 above, gives a description of the drugs that were prescribed. From this table we are able to understand that in our population sample 51.6% prescriptions had >8 drugs including a combination and two injections were prescribed in 50.3% of the sample.

**TABLE 2:** Types of drug related problems noted (Drug utilization study of diabetes and hypertension at tertiary care hospitals, India, five years study, 2020).

Type of drug choice problems	Frequency	Valid Percent
Inappropriate drug form	117	24.7
Inappropriate drug	89	18.8
No clear indication for drug use	88	18.6
Contraindication for drug	79	16.7
No drug prescribed but clear indication	72	15.2
Duplication of a therapeutic group or active ingredient	28	5.9
Total (1,914)	473	100.0
**Type of dosing choice problem**	**Frequency**	**Valid Percent**
Drug dose too high or dosage regime too frequent	159	33.9
Duration of treatment too short	141	30.1
Drug dose too low or dosage regime not frequent enough	98	20.9
Duration of treatment too long	71	15.1
Total (1,914)	469	100.0
**Compliance status**	**Frequency**	**Percent**
Poor compliance	914	47.8
Non-compliance	503	26.3
No direction	345	18.0
Good compliance	132	6.9
Unknown/not specified	20	1.0
Total	1,914	100.0

From Table 2 above, we are able to understand that inappropriate drug form, too high drug dose and poor compliance were the majorly responsible for drug regulated problems (24.7%, 33.9%, and 47.8%), respectively.

**TABLE 3:** Descriptive statistics: Overall cost of the treatment and length of stay in days (Drug utilization study of diabetes and hypertension at tertiary care hospitals, India, five years study, 2020).

Variable	Number of generic drugs prescribed	Mean	Std. Deviation	N
Overall cost of the treatment in INR	No generic drugs prescribed	21949.4	5386.3	156
	One drug only	23705.1	7016.4	212
	Two drugs combination	23948.9	7355.0	246
	Three drugs combination	22450.3	4993.4	371
	Four drugs combination	22154.3	5069.4	438
	Five drugs combination	22509.7	5379.8	491
	Total	22688.6	5767.4	1,914
length of stay in days	No generic drugs prescribed	6.3	2.4	156
	One drug only	6.6	2.4	212
	Two drugs combination	6.2	2.1	246
	Three drugs combination	6.3	2.2	371
	Four drugs combination	6.4	2.3	438
	Five drugs combination	6.5	2.3	491
	Total	6.4	2.3	1,914

The descriptive statistics of the overall cost of treatment and length of stay in days related to the number of generic drugs prescribed show that maximum treatment cost was observed (M = 23948.9, SD = 7355.0) INR 246 prescriptions when a combination of two drugs was given. The lowest treatment cost was observed (M = 21949.4, SD = 5386.3) INR when no generic drugs were prescribed. The maximum length of stay was observed (M = 6.6. SD = 2.4) days in 212 prescriptions when only one drug was given. The shortest LOS (M = 6.2, SD = 2.1) days were observed when the combination of two drugs was given.

**TABLE 4:** Parameter estimates: DV- Number of generic drugs prescribed (Drug utilization study of diabetes and hypertension at tertiary care hospitals, India, five years study, 2020).

Dependent variable	Parameter	B	Std. Error	t	*Sig.*
Overall cost of the treatment	Intercept	22509.7	258.8	86.95	*0.001*
	[Generic No = 0]	−560.2	527.1	−1.06	*0.288*
	[Generic No = 1]	1195.4	471.3	2.53	*0.011*
	[Generic No = 2]	1439.2	448.0	3.21	*0.001*
	[Generic No = 3]	−59.3	394.5	−0.15	*0.880*
	[Generic No = 4]	−355.3	376.9	−0.94	*0.346*
	[Generic No = 5]	0^a^	—	—	—
length of stay in days	Intercept	6.51	0.10	62.54	*0.001*
	[Generic No = 0]	−0.15	0.21	−0.72	*0.467*
	[Generic No = 1]	0.15	0.19	0.80	*0.423*
	[Generic No = 2]	−0.30	0.18	−1.69	*0.090*
	[Generic No = 3]	−0.12	0.15	−0.75	*0.451*
	[Generic No = 4]	−0.10	0.15	−0.65	*0.510*
	[Generic No = 5]	0^a^	—	—	—

^a^ This parameter is set to zero because it is redundant.

The mean number of generic drugs prescribed was found to be 3.15 whereas the maximum drugs was found to be five. The mean number of antibiotics prescribed was found to be 1.10 whereas the maximum number of antibiotics prescribed was found to be four. On evaluation of number of injections prescribed it was observed that the mean number of injections prescribed was found 2.17 whereas the maximum number of injections prescribed was found to be five in a day. The mean number of drugs prescribed from essential drug list (EDL) 2.33 whereas the maximum number of drugs prescribed from essential drug list was found five.

### Statistics of the Drugs Prescribed During Study

Supplementary File S1 demonstrates how the number of generic drugs prescribed showed that maximum times five drugs were prescribed in 491 patients followed by four drugs in 438 patients.

### Brief Summary of the Statistical Analysis We Conducted

Multivariate test was applied to compare no. of generic drugs prescribed with overall cost of treatment and length of stay.

Levene’s test of equality of error was found significant as *p*-value was >0.03 which assumes equality has been maintained between the samples.

The table test of between. subject effects shows that both the independent variable, length of stay and overall cost of treatment was found significant as *p*-value was found to be *p =* 0.001 and *p =* 0.030, respectively.

Cost is affecting more as compared to length of stay as *p*-value was found to be 0.001 and 0.030, respectively.

Overall cost: F (5, 1913) 5.2, *p* = 0.001, Length of stay: F (5, 1913) 1.0, *p* = 0.030.

The parameter estimates describe changes occurs in overall cost of treatment and length of stay.

The data reveals that the cost of treatment increased by *β* = 1439.2 for the patient prescribed 2 generic drugs as compared to above 5 drugs and *p*-value was found 0.001 and 0.011, respectively.

Supplementary File S2 has been provided which reveals that the number of genetic drugs prescribed as dependent variable shows significant results for overall cost of treatment F (5, 1908) = 5.2, *p* = 0.001 and length of stay in days F (5, 1908) = 1, *p* = 0.030.

The parameter estimates table for a dependent variable number of generic drugs prescribed shows that [Generic No = 5] as standard for overall cost of the treatment and length of stay (L.O.S) in days. The data revealed that the overall cost of treatment could be lower by *β* = −355.3 INR if four generic drugs were prescribed instead of five, followed by an increase in overall treatment cost were observed for two generic drugs by *β* = 1439.2 INR. It was also found that length of stay in days is not affected as much. In most of the groups the LOS was lowered by −0.10 to −0.30 days as shown in Table 4.

Supplementary File S3 has been provided which gives a summary of the drug related problems seen in our study (Punjab, India. 2020).

## Discussion

Embracing clinical pharmacy in treatment of patients has shown to be effective particularly in minimizing the economic burden and development of adverse drug reactions. Pharmacist interventions within a multi-disciplinary team have proven to be vital especially in conducting drug reviews. Drug review is a major component of pharmacy practice and it involves assessment of the medications given to patients and their possible outcomes (intended or unintended) (25, 26).

Inappropriate drug form and dose being too high were the main drug related problems discovered in our study. Another study by Rasool et al (27) showed a similar outcome with inappropriate drug from being responsible for 39.75% of the medication errors seen.

**FIGURE 2:**
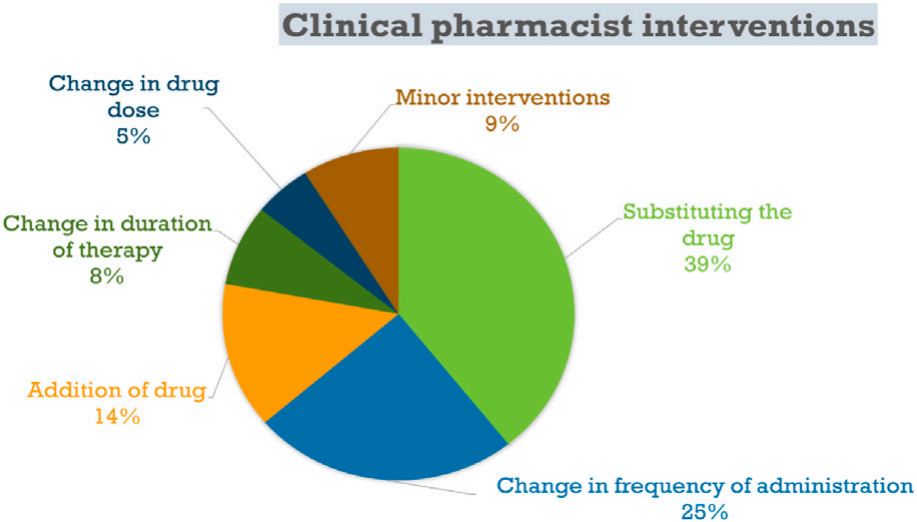
Summary of the clinical pharmacist interventions seen in our study (Drug utilization study of diabetes and hypertension at tertiary care hospitals, India, five years study, 2020).

A trial conducted by Sosale et al showed that 46% of patients diagnosed with type 2 diabetes mellitus are under the age of 40 (mean age was 34.7 ± 4.2 years) whereas the mean age of our study population was 54 years (28). The reason for this discordance in the age may be due to the different aims of the studies. Their study was tailored towards determining the onset of T2DM in young patients whereas our study had no specific target age group. Drug interactions were seen in 84.2% of our patients which was a little higher than the 70% which was seen in a study conducted by Sankar et al (29). Interestingly, their study only involved 50 patients which may not be able to give a proper representation of the larger population and hence this small difference. A study by Zazuli et al showed that inappropriate drug selection and inappropriate dosage selection contributed 25.3% and 4.4%, respectively occurrence of drug related problems. In our study, inappropriate drug form and inappropriate drug given contributed 6.11% and 4.65%, respectively in the 1,914 cases. Additionally, substituting the drug was the main intervention given by clinical pharmacists as seen in Figure 2. In their study, drug choice problem and dosing choice problem contributed 88.2% towards drug related problems (DRPs) whereas it only contributed 49.2% in our study. This was due to the fact their study was a prospective study for 3 months. In our study, it was revealed that patient compliance status was the most significant factor influencing the DRPs (30).

Our study revealed that pharmacists play a major role especially in patient follow-up and that patient compliance is very critical in ensuring desired therapeutic outcome. Also, as seen from other studies proper counselling, patient education and creating public health awareness improves the patients’ compliance (31). Some studies have shown a very good compliance rate of DM with HTN patients such as one conducted by Rao et al where 83.6% of the diabetics were compliant to their medications (32). Poor compliance and non-compliance were discovered to be the most prevalent medication related problem in our patients as they contributed 47.3% and 26.3%, respectively. A recent study has called on the National Programme for Control of Cancer, Diabetes, Cardiovascular Diseases, and Stroke (NPCDCS) in India to conduct more health awareness programmes especially in Punjab where there is a high rate of NCDs (Non-Communicable Diseases) (33). Multidisciplinary collaboration among the healthcare workers such as nurses, pharmacists and physicians has also proven to be a better means of achieving better patient care (34). This study is the first of its kind in North India because the sample size used in this study can be used to give an insight into the factors that are responsible for drug related problems in DM with HTN patients. This study was a 5-year study hence the results have a better representation generally of the kind of interventions involving pharmacists within our region. Furthermore, our study reveals the need for a more collaborative approach in medication treatment with the clinical pharmacist expected to play a great role in patient education as well as counselling.

### Limitation of Our Study

The study was limited to 3 hospitals only. In North India, many hospitals exist and similar study needs to be conducted for a better overview.

### Impact of Findings on Practice

• Clinical pharmacists are an asset to the healthcare team and patients.
• Most of the drug related problems can be averted through proper patient counselling.
• Patient compliance should not be overlooked.

### Conclusion

Our study concludes that pharmacist’s intervention is critical in the improvement of health outcomes in DM with HTN patients. Moreover, our study revealed that patient compliance was found as the greatest contributor to drug related problems.

## Abbreviations

DBP, diastolic blood pressure; DM, Diabetes mellitus; DRPs, drug related problems; FBS, fasting blood sugar; HTN, hypertension; INR, Indian rupees; RBS, random blood sugar; SBP, systolic blood pressure; USD, United States dollar.
