# Supplementary material for: Pharmacist Interventions in Minimizing Drug Related Problems in Diabetes With Co-Existing Hypertension: A Five-Year Overview and Ground Report From India
Source: Int J Public Health. 2023 Apr 3;68:1605808. doi: 10.3389/ijph.2023.1605808 (PMC10106567; doi:10.3389/ijph.2023.1605808)

Supplementary files

Supplementary file 1: Between-subject's factors: Number of generic drugs prescribed (Drug utilization study of diabetes and hypertension at tertiary care hospitals, India, five years study.)

Variable	SPSS Code	N
Number of generic drugs prescribed	0	156
	1	212
	2	246
	3	371
	4	438
	5	491

Supplementary file 2: Tests of between-subject's effects: DV- Number of generic drugs prescribed (Drug utilization study of diabetes and hypertension at tertiary care hospitals, India, five years study.)

Source	Dependent Variable	Type III Sum of Squares	df	Mean Square	F	*Sig.*
Corrected Model	Overall cost of the treatment	856825932.4[a]	5	171365186.4	5.2	*.001*
	Length of stay in days	28.7[b]	5	5.7	1.0	*.30*
Intercept	Overall cost of the treatment	841690771174.0	1	841690771174.0	25581.8	*.001*
	Length of stay in days	66923.9	1	66923.9	12566.8	*.001*
Generic No.	Overall cost of the treatment	856825932.4	5	171365186.4	5.2	*.001*
	Length of stay in days	28.7	5	5.7	1.0	*.030*
Error	Overall cost of the treatment	62776750847.1	1908	32901861.0		
	Length of stay in days	10160.9	1908	5.3		
Total	Overall cost of the treatment	1048910254130.0	1914			
	Length of stay in days	89375.0	1914			
Corrected Total	Overall cost of the treatment	63633576779.6	1913			
	Length of stay in days	10189.6	1913			

a. R Squared = .013 (Adjusted R Squared = .011)
b. R Squared = .003 (Adjusted R Squared = .001)

Supplementary file 3: Summary of the drug related problems seen in our study (Drug utilization study of diabetes and hypertension at tertiary care hospitals, India, five years study.)

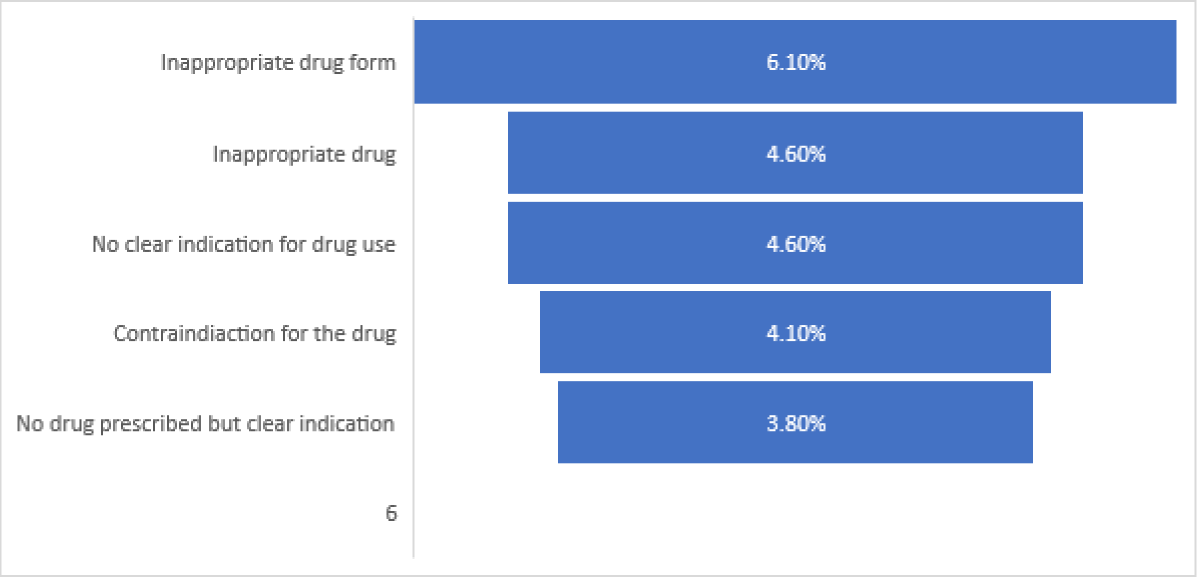

Supplement: Supplementary file 1 [file DataSheet1.pdf]
